# Combining Sound and Deep Neural Networks for the Measurement of Jump Height in Sports Science

**DOI:** 10.3390/s24113505

**Published:** 2024-05-29

**Authors:** Lucas Banchero, Jose J. Lopez, Basilio Pueo, Jose M. Jimenez-Olmedo

**Affiliations:** 1 Institute of Telecommunications and Multimedia Applications, Universitat Politecnica de Valencia, 46022 Valencia, Spain; lbanmar@upv.edu.es; 2 University Institute for Computing Research, University of Alicante, 03690 Alicante, Spain; basilio@ua.es; 3 Research Group in Health, Physical Activity, and Sports Technology, University of Alicante, 03690 Alicante, Spain; j.olmedo@ua.es

**Keywords:** instrument, validation, accuracy, test, reliability, robustness, signal, detection, CNN

## Abstract

Jump height tests are employed to measure lower-limb muscle power of athletic and non-athletic populations. The most popular instruments for this purpose are jump mats and, in recent years, smartphone apps, which compute jump height through the manual annotation of video recordings and recently automatically using the sound produced during the jump to extract the flight time. In a previous work, the afore-mentioned sound systems were presented by the authors in which the take-off and landing events from the audio recordings of jump executions were obtained using classical signal processing. In this work, a more precise, noise-immune, and robust system, capable of working in the most unfavorable environments, is presented. The system uses a deep neural network trained specifically for this purpose. More than 300 jumps were recorded to train and validate the network performance. The ground truth was a jump mat, providing a slightly better accuracy in quiet and medium quiet environments but excellent accuracy in noisy and complicated ones. The developed audio-based system is a trustworthy instrument for measuring jump height accurately in any kind of environment, providing a perfect measurement tool that can be accessed through a mobile phone in the form of an app.

## 1. Introduction

Vertical jump height serves as a fundamental metric for evaluating the muscular strength and coordination of the lower extremities [1,2], providing valuable insights for athletes and individuals engaging in physical activities. This measure is widely utilized by sports professionals to monitor the neuromuscular and performance characteristics of both athletes and non-athletes. Any changes in jump height are indicative of shifts in functional performance [3].

There are a variety of tools and methods available for evaluating lower body power through vertical jump tests. One approach involves the use of force plates to measure ground reaction forces, which can then be numerically integrated to determine jump height [4]. Another method involves tracking the body’s center of gravity during a jump using biomechanical motion capture, which allows for the analysis of movement patterns [5,6]. Lastly, the duration between take-off and landing can be converted into jump height using basic linear kinematics, with timekeeping instruments used to measure the flight times of athletes [7].

Timekeeping instruments have gained popularity in the sports world due to their simplicity, portability, and affordability compared to lab equipment, like force plates and motion capture systems. These instruments accurately select take-off and landing time events to calculate the displacement of the center of gravity during flight or jump height [8]. The first of these were jump mats, or contact mats, which function as an electric switch activated by the athlete’s weight [9]. Later, photocell mats were developed, featuring an array of IR diodes that create an optical barrier interrupted by athletes during jumps [10,11]. More recently, smartphone apps have emerged that use high-speed video recordings to allow users to manually select take-off and landing frames. The flight time is then computed by counting the number of frames between these events [12]. These apps have become prevalent in sports sciences due to the inherent advantages of smartphones, such as portability, connectivity, and data processing capabilities.

While the estimation of jump height through flight time is a proven method [9], the manual digitization of events by video observation presents several challenges. The image sensor’s sampling frequency in smartphones is often insufficient for the temporal resolution needed to select key frames. Additionally, the take-off and landing phases of a vertical jump, which exhibit the maximum velocity values, are typically undersampled in slow-motion videos [13]. Shutter speeds, which are usually not user-operable and only reach their fastest speed in brightly lit scenes, can result in slightly blurred images of feet during the take-off and landing phases in most indoor or poorly lit environments [14]. These factors can affect measurement accuracy due to uncertainty in selecting the correct frame. For example, an observation inaccuracy of just one frame in the take-off and landing events can introduce an error of 0.9 cm for a 30 cm jump when using a high-speed video of 240 fps. Furthermore, different observers may yield different results due to varying precision levels and potential bias, known as the observer effect [15]. The manual analysis required by these systems also lacks the necessary speed for sessions involving a large number of athletes. As such, apps based on manual digitization by video observation have several limitations that prevent them from being valid alternatives to other instruments like jump mats.

A previous investigation proposed a novel audio-based system that automatically detected take-off and landing moments during jumps, outperforming high-speed video smartphones in time precision [16]. While this system demonstrated excellent precision in quiet and moderately quiet environments, it was found to detect false positives in highly acoustically challenging environments. These false positives corresponded to non-executed jumps or, at times, small errors when an impulsive noise coincided closely with the sounds of the jump.

In this paper, we developed a highly accurate algorithm designed to operate effectively in diverse acoustic environments. Our objective was to create a robust, unattended commercial product usable in any environmental condition without constraints. To attain this goal, a deep neural network was trained using take-off and landing sounds, effectively mitigating confusion with other impulsive environmental sounds.

## 2. Methodology and Preprocessing

### 2.1. Experimental Procedure

**Figure 1:**
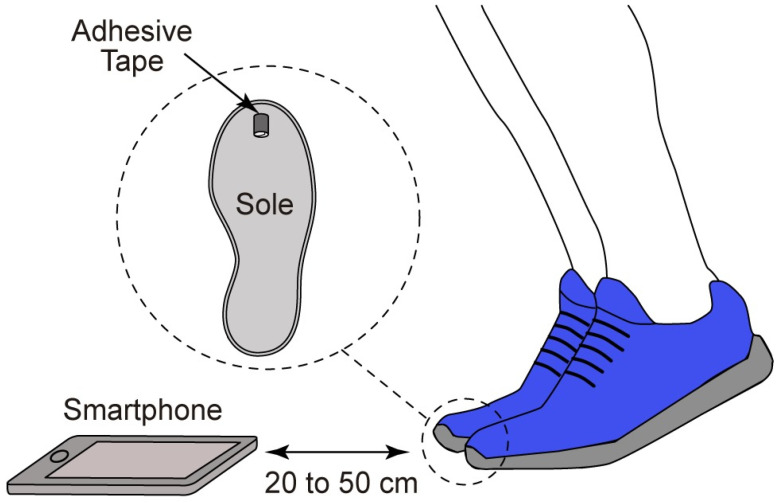
Experimental setup of jump recordings with the microphone of a smartphone.

An audio capture system was used to record the sound wave produced by an athlete during a vertical jump. Our objective was the automatic extraction of the take-off and landing phases. The microphone was strategically placed to capture audio waveforms from both feet at these critical moments. Similarly to what was conducted in [16], we employed a piece of adhesive tape stuck to the sole of the shoe for identifying take-off. All measurements were conducted using a smartphone’s audio system to replicate conditions similar to manual video digitization through smartphone apps, as shown in Figure 1. The selected smartphone operated at a sampling frequency of 48,000 Hz and the raw audio signals were subsequently transferred to a computer for in-depth analysis.

To assess accuracy, all jumps were measured concurrently using a validated jump mat system (Chronojump-Boscosystem, Barcelona, Spain) as a benchmark [17]. Subsequently, after algorithm development, a study of repeated measurements of countermovement jump (CMJ) height during a single test session was carried out. The session commenced with a standardized warm-up, including 5 min of running with directional changes and 3 min of dynamic stretching and movement exercises. Following this, the subjects received instruction and practice in proper CMJ execution for 2 min. Finally, the subjects performed three CMJ [3] with a one-minute rest between the trials. The executions were meticulously monitored for precise technique, and only successful trials were considered. The subjects were instructed to execute jumps at varying heights, encompassing a comprehensive range of jump height measurements.

### 2.2. Audio Signal Characteristics of the Jumps

In our previous work [16], it was demonstrated that the algorithm based on classical signal processing techniques yielded excellent results, even in moderately noisy environments. Realistic samples were included in the study, taken from gyms and sports facilities, albeit not in particularly challenging situations such as crowded sports arenas or gyms during moments of intense impulsive noise, like weight dropping or noise generated by training equipment. Additionally, outdoor samples were not taken. The objective of the current study was to develop a much more robust algorithm in high background noise situations, as mentioned previously, while being equally robust and accurate as the classic algorithm in quieter environments.

**Figure 2:**
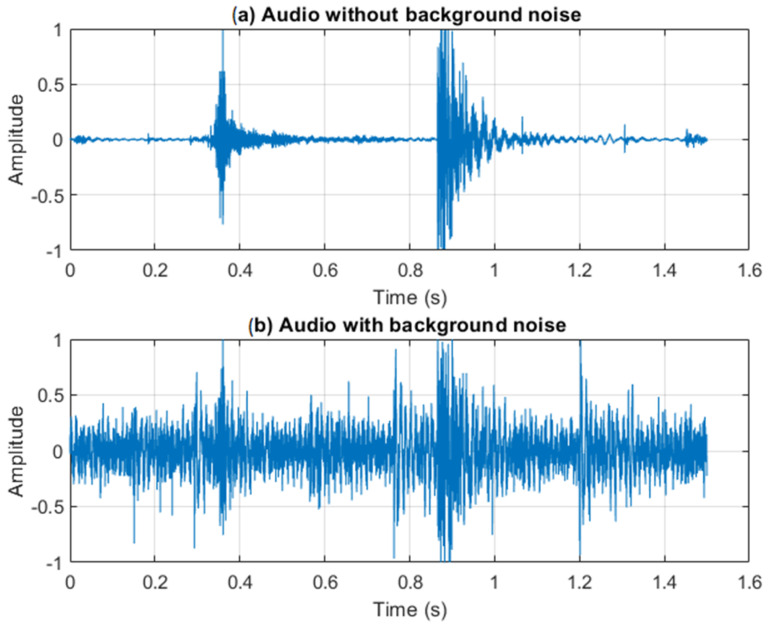
Typical jump audio signal showing take-off and landing events: (**a**) without background noise; (**b**) with background noise.

Figure 2a depicts a jump signal in a moderately noisy environment similar to the database used in the previous work. Slight background noise and occasional impulsive noises can be observed. In contrast, Figure 2b illustrates the same jump in a highly noisy environment, where identifying take-off and landing events becomes significantly more challenging. During training sessions, especially in indoor facilities, background noise levels may range from 70 to 90 dBA. Decibels (dBA) represent a logarithmic scale used to express the intensity or loudness of sound, with each 10 dB increase indicating a tenfold increase in sound intensity. This noise can come from various sources, such as equipment (e.g., weights and machines), coaches providing instructions, and athletes communicating with each other. The nature of noises in such environments is diverse and is a function of the facilities, equipment, number of people present, and the type of activities being performed. However, this noise can be generally modeled as a combination of a constant background noise level, interspersed with impulsive noises from various sources, such as weight dropping, machines clanking, and other sudden impact sounds. The take in Figure 2b was recorded in a crowded sports arena where the background noise was 80 dBA SPL, measured with a sound meter. Moreover, the additional interferences in this take, especially from impulsive sounds, pose a significant challenge to the classical algorithm as it can mask the distinctive patterns associated with take-off and landing events. The previous algorithm relied on adaptative energy levels, but such a method proved ineffective in the presence of background noise and impulsive sounds, as will be demonstrated in Section 4.

Considering the analysis of the samples we encountered, such as the one depicted in Figure 2b, the objective of this work was to develop an AI-based algorithm that enables us to measure flight time by discerning between interferences and genuine jump events effectively. Our approach focused on developing techniques to mitigate the effects of background noise and impulsive sounds on jump event detection.

### 2.3. Addition of Background Noise and Interferences

As previously mentioned, the noise in highly noisy environments, such as crowded sports arenas and gyms, can be modeled as a combination of constant background noise and interspersed impulsive noises. To enhance the detection algorithm and make it more robust in such challenging conditions, we considered signals of jump landing and take-off sounds mixed with highly noisy environmental recordings. By artificially introducing these jump sounds into real-world noise samples, we can systematically evaluate the performance of our improved algorithm under controlled yet realistic conditions.

**Figure 3:**
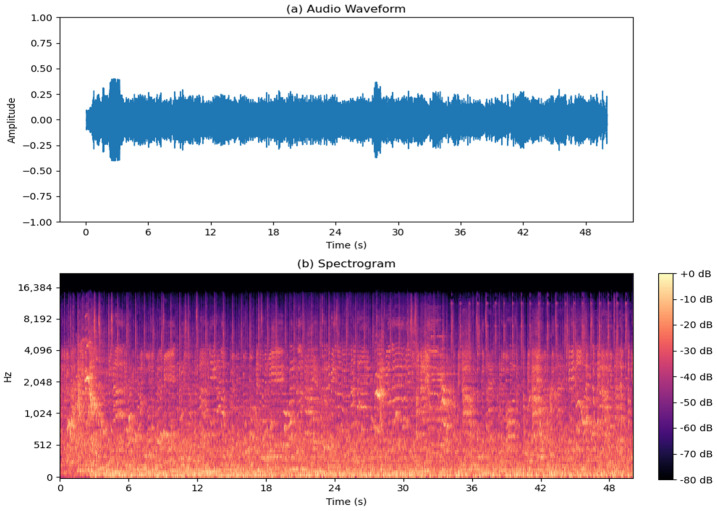
Typical background noise: (**a**) waveform; (**b**) spectrogram.

Background noise from training sessions typically ranges from 70 to 90 dBA and forms the constant hum of activity within these spaces. This ambient noise encompasses the collective sounds of equipment operation, athlete communication, and environmental factors. The first signal encapsulated solely steady background noise, mirroring the ambient audio environment typically observed in training session facilities (Figure 3). The typical background noise is composed of music, murmurs, and people’s voices engaged in sports activities, all with a significant reverberation to emulate typical gymnasium environments. As depicted in the figure, the energy of these events is concentrated in the low frequencies. As discussed earlier, having resolution in these frequencies allows the AI to better discern the correct sounds of interest.

**Figure 4:**
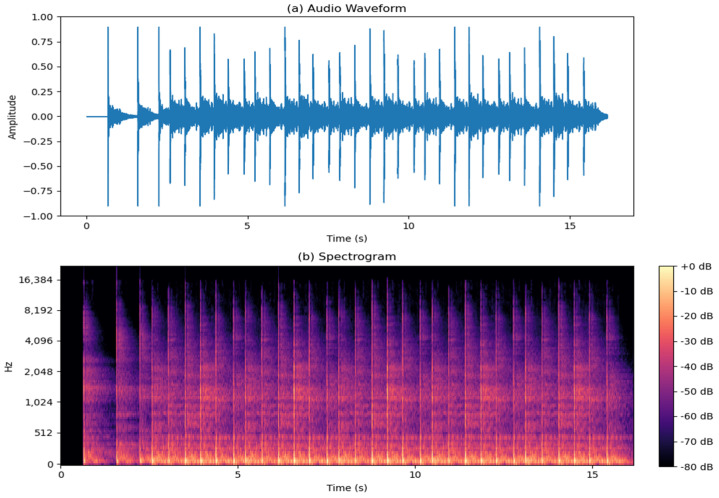
Typical impulsive noise: (**a**) waveform; (**b**) spectrogram.

Figure 4 shows the second signal-integrated impulsive noises that are characteristic of sport facilities. These impulses, interspersed within the steady background noise, mirrored the abrupt sound bursts generated during athletic activities. This signal contains impulsive noises, such as impacts and, notably, the sound of bouncing balls, which is a typical impulsive sound in sports environments, which were also added. The spectrogram illustrates how this type of sound is distributed across the entire frequency spectrum.

The purpose of adding these noises is to contaminate the original audio samples across as many frequencies as possible, simulating real-world conditions. This noise, consisting of both background noise typical of noisy environments, approximately equivalent to 80 dBA SPL with a balanced distribution around the mobile device at a close distance from the feet, and impulsive noises, ensures that the AI model is trained to accurately discern relevant sounds amidst realistic acoustic environments. This comprehensive approach enhances the model’s ability to generalize and accurately distinguish between relevant sounds and background noise in diverse scenarios.

### 2.4. Feature Extraction

**Figure 5:**
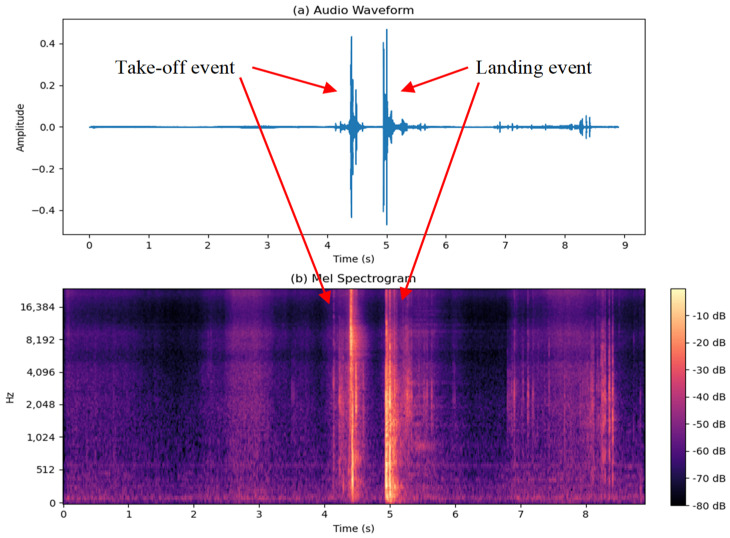
Typical jump event: (**a**) waveform; (**b**) Mel spectrogram.

The utilization of time-frequency parameters, such as spectrograms, in classification tasks employing convolutional neural networks (CNNs) is justified by their ability to capture both temporal and frequency information simultaneously. Spectrograms provide a comprehensive representation of the signal’s frequency content over time, enabling CNNs to discern intricate patterns and variations crucial for accurate classification. This approach enhances the network’s capacity to discriminate between different classes by leveraging the rich temporal dynamics and frequency characteristics present in the data, ultimately improving the classification performance. Furthermore, the utilization of Mel-frequency scale [18] in CNN-based classification tasks is particularly compelling and is more aligned with human auditory perception compared to linear frequency scales, offering a logarithm resolution in the frequency axis that has been proven very useful for dealing with sound signals. In this case, it is quite advantageous for capturing the characteristics of jump noise, as demonstrated in Figure 5. Jump events often exhibit energy in these low-frequency ranges, making Mel spectrograms effective in distinguishing between jump events and the prevalent background noise in noisy environments.

In this case, we calculated a Mel spectrogram with 128 bands employing a window size of 16.6 ms, equivalent to 800 samples, providing a fine temporal resolution while maintaining enough frequency resolution for our problem. An overlap of 50% between windows was chosen, with a hop length of 8.8 ms.

**Figure 6:**
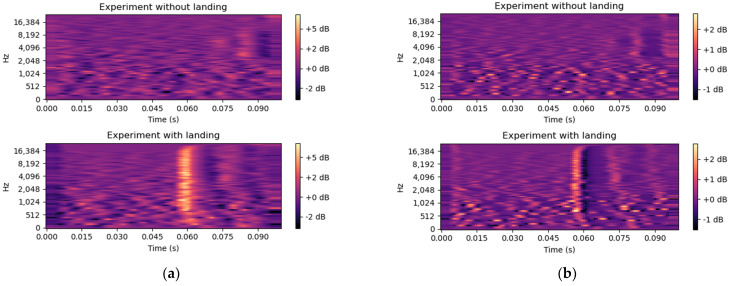
Comparison of Mel spectrograms for landing and non-landing events: (**a**) delta Mel spectrogram; (**b**) delta-delta Mel spectrogram.

To improve the detection of the acoustic events, more features were calculated to feed the neural network, as the delta spectrogram and delta–delta spectrogram features. They were extracted from the Mel spectrogram by processing differences [19,20]. These features not only provide energy information per frequency band but also capture the velocity and acceleration of audio changes, as it can be seen in Figure 6. Consequently, the regions where a landing or take-off event occurs exhibit more prominently excited delta and delta–delta features compared to other regions without these events, further aiding in the accurate detection of jump noise.

### 2.5. Data Preparation and Labeling

To train a DNN (deep neural network) effectively, it is essential to have a correctly labeled training dataset, which serves as the ground truth. Our dataset comprised 300 audio recordings sourced from a previous paper [16], wherein precise landing and take-off events were already identified and time-labeled. Using data augmentation based on combining with the noise addition process explained in Section 2.3, we created a dataset of 600 samples that was employed to train and verify the DNN, as explained in Section 3.3.

Once the Mel spectrograms, along with their corresponding delta and delta–delta features, were obtained, as explained in the preceding section, the data underwent preparatory steps for training. Similar to how an RGB image has three dimensions (red, green, and blue), we adopted an approach by concatenating the Mel spectrogram, its delta, and its delta–delta features in the third dimension of the tensor, effectively creating three dimensions alongside the dimensions of time and frequency inherent to spectrograms. This process enhances the representation of temporal and frequency features within each sample, providing the neural network with richer information to learn from.

In tackling the problem at hand, the objective was to detect the occurrence and temporal position of the jump event within a temporal segment containing spectrogram data. Directly inputting the entire temporal segment into the neural network would pose inefficiencies, as it would overlook temporal information crucial for pinpointing the event’s occurrence. To address this issue, a strategy was needed to selectively feed segments of the spectrogram data into the network at intervals, allowing for both computational efficiency and the temporal localization of the event.

To achieve this, a compromise must be struck between the temporal resolution of the input segments and the computational burden imposed. Introducing segments at too fine a temporal resolution, determined by the hop length, could overwhelm the computational resources, while too coarse a resolution might sacrifice temporal precision in event localization. Thus, a judicious selection of both the temporal separation between input segments and their size is essential.

The landing and take-off events last between 50 and 80 ms. Therefore, the segment of the spectrogram that should be used as input to the neural network must contain at least this temporal value. After several initial attempts, it was decided to use a fragment of 100 ms as input to the CNN, which corresponds to 12 spectrogram windows totaling approximately this duration. This choice ensures that take-off and landing events are contained within each segment. The specific duration was chosen to strike a balance between the computational efficiency and ensuring that each window encapsulates an entire event. Subsequently, the segmented spectrogram was annotated employing the commented database at the beginning, with a binary value assigned: 1 indicates the presence of a landing event within the window, whereas 0 signifies its absence. The same was carried out for the take-off events.

## 3. Event Detection Using CNN

### 3.1. Introduction to CNNs

Convolutional neural networks (CNNs) are a specialized type of neural networks known for their ability to extract features and automatically learn complex and significant patterns from a given dataset [21]. These networks consist of multiple layers of interconnected neurons that learn to extract hierarchical features from input data through convolutional operations, pooling, and non-linear activation functions. By leveraging these hierarchical features, CNNs can effectively learn complex patterns and relationships. While CNNs have primarily been applied to image analysis, they have increasingly demonstrated their high efficiency in audio classification and detection tasks [18].

One important aspect of CNNs in audio event detection is their capability to learn in a non-linear and hierarchical manner [22]. This means that these networks can acquire low-level audio features, such as spectral characteristics and frequency components, and combine them to form higher-level features [23]. This hierarchical learning capacity allows CNNs to capture more abstract representations of the audio data, making them especially valuable in the detection of audio events [24]. CNNs can identify key audio features and establish relevant temporal and spectral relationships associated with specific audio events. For this reason, CNNs have been used to detect fall events provided by audio, thus addressing the limitations of the previous algorithm.

### 3.2. CNN Architecture

**Figure 7:**
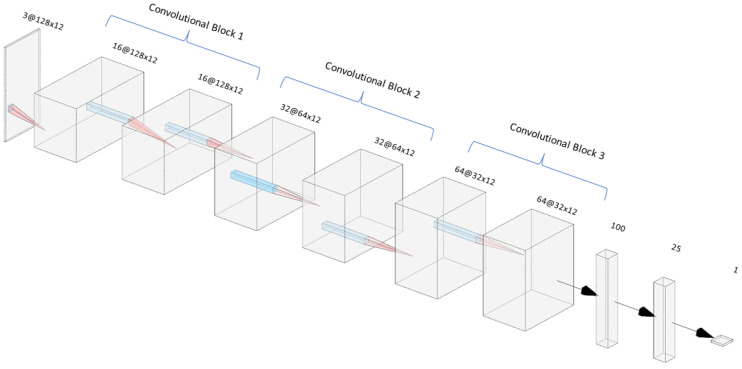
AI model architecture.

As seen in the previous section and based on experience with similar classification problems, we propose the following model architecture: The model is built using convolutional blocks, each of which has a basic architecture of three 2D convolutional layers, along with activation, normalization, dropout, and 2D pooling layers. At the end of these blocks, a flatten layer is implemented, followed by a couple of dense layers that analyze the features extracted by the convolutional networks. To finalize the model, a final output layer with sigmoid activation is added, providing binary classification, 0 or 1, depending on the event that is detected. The overall structure of the network is shown in Figure 7.

In each convolutional block, the model is sequentially divided into a series of layers optimized for feature extraction and dimensionality reduction. It begins with a Conv2D layer with ReLU activation, which performs a 2-dimensional convolution operation on the input feature maps, extracting features using learnable filters while introducing non-linearity through ReLU activation. This is followed by batch normalization, a layer that normalizes the activations of the previous convolutional layer across the batch dimension, stabilizing and accelerating the training process. Another Conv2D layer with ReLU activation is then applied, performing another 2-dimensional convolution operation to further extract higher-level features from the input feature maps while maintaining non-linearity through ReLU activation. Subsequently, a dropout layer with a rate of 0.25 is introduced to mitigate overfitting by randomly deactivating a fraction of neurons during training, promoting the robustness and generalization of the model. Finally, an average pooling layer is applied to reduce the spatial dimensions of the feature maps, aiding in feature extraction and computational efficiency by downsampling the feature maps. As shown in Figure 7, in each convolutional block, the number of filters is doubled as the signal becomes more complex, starting with 16, then 32, and finally 64 filters. At the output of the last convolutional block, a flatten layer is applied, which serves as the input for the fully connected layers. The fully connected layers consist of a PReLU activation layer, followed by a dropout layer and batch normalization. Subsequently, there is another layer with linear activation. Lastly, an output layer with a single neuron and sigmoid activation is added, as mentioned before. Overall, the model has 900,000 neurons.

### 3.3. Model Training

Once the model architecture was structured, we commenced the training phase. Given that the sound level at landing, indicative of the impact of feet on the floor, is significantly louder than that at take-off, it is reasonable to utilize landing as a triggering event to identify a jump execution. Consequently, upon estimating the time position of the landing, the algorithm proceeds to search for the take-off in a preceding position. This approach enhances efficiency by training a binary neural network specifically to detect landings, which continuously explores the temporal signal until detection. Subsequently, upon detection, a second network is engaged to identify take-offs only within the possible temporal window preceding a landing, thereby saving computational cost.

Therefore, instead of training a network to detect three classes—landings, falls, and nothing—we opted to train two binary networks, each independently detecting landings and take-offs. This approach mitigates the risk of increased error rates due to potential false positives, a scenario more likely to occur when working with three classes. For each network, spectrograms of the 0.1 s segments serve as the input features during training, with the binary label indicating the presence (1) or absence (0) of the landing or take-off event, respectively.

The training process comprised 100 epochs with a batch size of 64, incorporating early stopping with a patience of 10. Precision was selected as the monitored metric, given its importance in minimizing false positives and reducing potential errors in landing detection within the audio recordings. The training dataset constituted 80% of the total data, with 10% allocated for testing and the remaining 10% reserved for the evaluation of the final system. Although precision was monitored for early stopping, the recall and F1-score metrics were utilized to evaluate the model’s performance applied to the final evaluation samples. Given the binary classification nature of the problem, the model was compiled with binary cross–entropy loss and the Adam optimizer, employing a learning rate of 0.01. To assess the model’s generalization ability, a 5-fold cross-validation was implemented.

Cross-validation is a widely employed technique in machine learning for assessing the generalization performance of a model. It involves partitioning the dataset into complementary subsets, performing training and validation iteratively on different subsets, and then averaging the results to obtain a robust estimation of the model’s performance. In the context of this study, cross-validation is particularly beneficial for evaluating the model’s ability to generalize to unseen data while mitigating the risk of overfitting. By dividing the dataset into multiple folds and training the model on different combinations of training and validation sets, cross-validation provides a more comprehensive understanding of the model’s performance across various data distributions. This approach helps to ensure that the model’s performance metrics, such as precision, recall, and F1-score, are robust and representative of its true capabilities when deployed in real-world scenarios. While there exist other, more complex validation techniques [25,26], for the current scope of our article, we believe our validation approach may serve our purposes adequately.

For this purpose, a program was developed in TensorFlow to facilitate the implementation of the neural network architectures and the training process. TensorFlow provides a comprehensive framework for building and training deep learning models efficiently. The program is structured to handle the entire pipeline, from data preprocessing to model evaluation. Additionally, the program incorporates functionalities for model configuration, including defining the network architecture, selecting optimization algorithms, and setting hyperparameters.

Furthermore, the program supports early stopping mechanisms to prevent overfitting and save computational resources. Once the training process is complete, the program facilitates model evaluation using separate validation datasets and generates performance metrics and visualizations for analysis. Overall, the TensorFlow program streamlines the development and training of neural network models for jump noise detection, providing a scalable and efficient solution for researchers and practitioners in the field.

### 3.4. Training Results

Tackling the complexities of background noise presented a formidable challenge throughout our training efforts. The pervasive nature of background noise, especially in masking low-frequency components, alongside impulsive noises overshadowing high-frequency signals, posed significant hurdles during the training process. Despite these daunting obstacles, considerable efforts were directed towards devising strategies to mitigate the adverse effects of background noise and impulsive interferences on model performance.

To assess the quality of our predictions, we utilized precision, recall, and F1-score metrics. These metrics were chosen deliberately due to the imbalance in class distribution within our dataset. In the context of Mel spectrograms divided into 0.1 s fragments, it is far more common to encounter segments labeled as 0, indicating the absence of any event, compared to segments labeled as 1, where a landing or take-off event occurs. Therefore, accuracy alone would not provide an accurate representation of the model’s performance. The precision, recall, and F1-score metrics are well-suited for evaluating the performance of our model in such scenarios.

Precision, as described in (1), measures the proportion of true positive predictions among all positive predictions made by the model. It indicates the model’s ability to correctly identify positive instances without misclassifying negative instances as positive. A high precision value indicates that the model produces few false positives, making it valuable in scenarios where false positives are costly or undesirable.

(1) $$\mathrm{Precision}=\frac{\mathrm{True}\;\mathrm{Positives}\;}{\mathrm{True}\;\mathrm{Positives}+\mathrm{False}\;\mathrm{Positives}},$$

Recall, denoted as (2), also known as sensitivity or true positive rate, measures the proportion of true positive predictions among all actual positive instances in the dataset. It reflects the model’s ability to capture all positive instances, minimizing false negatives. High recall values suggest that the model effectively identifies most positive instances in the dataset, making it suitable for scenarios where missing positive instances is a significant concern.

(2) $$\mathrm{Recall}=\frac{\mathrm{True}\;\mathrm{Positives}\;}{\mathrm{True}\;\mathrm{Positives}+\mathrm{False}\;\mathrm{Negatives}},$$

The F1-score, referred to as (3), is the harmonic mean of precision and recall, providing a balanced assessment of the model’s performance. It takes into account both false positives and false negatives, making it a useful metric for evaluating classifiers with imbalanced datasets. A high F1-score indicates a balance between precision and recall, signifying a robust performance across both metrics.

(3) $$\text{F1-Score}=\frac{2\cdot \mathrm{Precision}\cdot \mathrm{Recall}}{\mathrm{Precision}+\mathrm{Recall}},$$

Our program demonstrated exceptional performance in binary classification for landing detection, as evidenced by the evaluation results presented in Table 1. Across all five iterations of k-fold cross-validation, our model consistently achieved high precision values, ranging from 0.925 to a perfect precision of 1.0. This indicates that the majority of predicted positive instances were indeed true positives, with very few false positives. Our model also achieved very good recall, with scores between 0.891 and a perfect 1.0. This means it effectively captured most of the relevant cases. The F1-score, a balanced measure of precision and recall, ranged from 0.943 to a perfect score of 1.0, highlighting the robustness of our model’s performance across different evaluation iterations. The mean values of precision (0.970), recall (0.963), and F1-score (0.966) further underscore the consistent and reliable performance of our program in accurately detecting jump noise events within audio segments. These results validate the effectiveness of our approach and confirm the utility of the precision, recall, and F1-score metrics in assessing the performance of classifiers operating on imbalanced datasets.

In addition to the high accurate performance showcased by our program in detecting landing events, similarly outstanding results were achieved by the counterpart AI responsible for identifying take-off events. These results, as demonstrated in Table 2, mirror the excellence observed in our model’s precision, recall, and F1-score metrics, affirming the robustness and efficacy of our approach across both phases of jump noise detection. The consistent attainment of high precision, recall, and F1-score values across five iterations of k-fold cross-validation underscores the reliability and generalizability of our models in real-world applications. By achieving comparable levels of accuracy in detecting take-off events, our comprehensive approach ensures a thorough and accurate identification of jump noise events throughout the entirety of audio segments. This synchronized performance across complementary models further strengthens the credibility and utility of our methodology in addressing the challenges posed by noisy and impulsive acoustic environments.

### 3.5. Time Flight Extraction

Now that we utilized CNNs to determine the temporal segments containing the take-off and landing events of a jump, the next step involved precisely calculating the flight time of the jump. This required additional signal processing calculations to extract the necessary temporal information.

Flight time, in the context of a jump, refers to the duration between take-off and landing. To compute this accurately, signal processing techniques, such as event detection and time stamping, are employed. This involves identifying specific features within the temporal segments corresponding to take-off and landing events, and then determining the exact timestamps associated with these features. Once the timestamps of both take-off and landing events are determined, the flight time can be calculated as the difference between these timestamps.

To ensure continuity, we now revisit the sequential process outlined earlier. Initially, the CNN is utilized to detect the landing event within the temporal segments. Subsequently, within a reasonable preceding temporal window, the take-off event is sought. The take-off event must fall within a reasonable timeframe, typically no more than 0.7 s, corresponding to human performance constraints [16]. This ensures the temporal coherence and physiological plausibility of the detected events within the context of human movement dynamics.

To determine the flight time, a precise localization process is implemented to acquire the exact onset of each event. The algorithm employed for this purpose entails an iterative analysis in time of signal energy. Initially, the signal’s energy distribution is computed and normalized to a scale ranging from 0 to 1, enabling consistent comparison across various segments and signals. Subsequently, a threshold, initially positioned near 1, is imposed on these normalized energy values to serve as a discriminant for event detection, where energy values exceeding the threshold signify event occurrences. As the algorithm progresses through the signal data, encountering energy values falling below the threshold, it dynamically adjusts the threshold downwards. This adaptive thresholding mechanism enables the algorithm to adapt to fluctuations in signal energy, ensuring accurate identification of event onsets.

## 4. Experiments and Algorithm Comparison

### 4.1. Introduction of the Experiments

Having successfully trained the neural network and observed its proficiency in detecting events, our primary objective shifted to evaluating the algorithm’s performance comprehensively. Specifically, we aimed to accurately determine the flight time between the jump and landing events. Unlike the binary classification of landing events, the flight time represents a continuous numerical value, serving as our ground truth for evaluation. In assessing the algorithm’s efficacy, we focused on minimizing errors in estimating the flight time compared to this ground truth, obtained from the jump mat experiments conducted in the previous paper [16]. In this section, we conduct various evaluations, comparing our algorithm’s performance against the classical algorithm presented in [16] when subjected to high-intensity noises, such as those introduced in Section 2.3.

In order to prevent the AI system from being biased towards the specific noise patterns present in the training data, the addition of noise to the clean samples was randomized once again. This involved introducing noise with similar characteristics but not identical to the noise added in the training samples of the CNN. This process aims to evaluate the generalization ability of our model. When validation is conducted using exactly the same samples as those used for training, there is a risk of the AI being biased and producing better results than it should.

In this section, our focus lies on a comprehensive comparison between the new and old algorithms, targeting key performance metrics. Firstly, we commence our analysis by scrutinizing false positives, given their pivotal role in the algorithm’s performance. This examination seeks to uncover any differences in false positive rates between the two algorithms, offering insights into their respective capabilities in accurately detecting landings in noisy environments. Following this, we proceed to compare flight time estimation between the algorithms. This evaluation step aims to discern any discrepancies in the accuracy of flight time estimation, the most critical aspect in jump event analysis. Finally, we employ the synthesis of new audio samples to assess the new algorithm’s capacity to generalize to unseen data. This evaluation scenario aims to gauge the robustness and adaptability of the new algorithm across diverse environmental conditions, offering insights into its potential applicability in real-world settings.

### 4.2. Analysis of Landing False Positives

As explained earlier in Section 3.3, given the sequential nature of the algorithm—first detecting the landing and then searching for take-off within a precise time window—the analysis of false positives for landings takes precedence. To ensure a fair comparison between the two algorithms, a false positive was defined as the algorithm detecting a landing outside a window of ±0.05 s, centered on the landing point. The reason for choosing ±0.05 s was because the spectrogram was divided into segments of 0.1 s. Therefore, a margin of 0.05 s before and after the exact landing point (which was determined using the ground truth) was left. If an event was detected outside this margin, it was considered a false positive.

**Figure 8:**
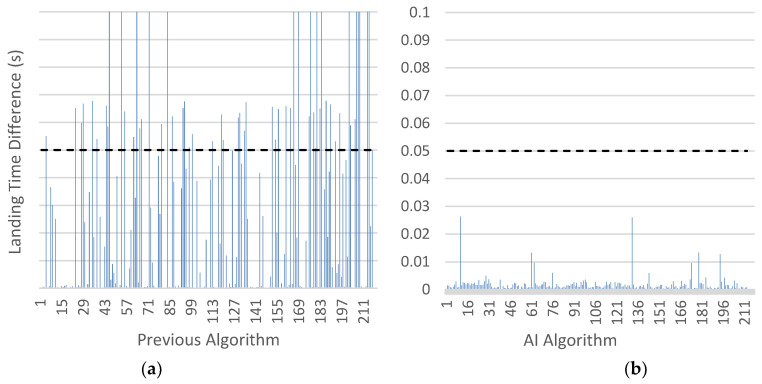
False positives in the algorithms. Blue line: time differences between ground truth and algorithm landing predictions: (**a**) previous algorithm; (**b**) AI algorithm.

Figure 8 illustrates the introduction of noise leads to the detection of additional points with low-frequency energy as jump landings in the old algorithm. This occurrence arises because the previous algorithm terminated upon detecting a value exceeding a threshold of low-frequency energy. The old algorithm exhibits 57 false positives out of a total of 216 validation samples, accounting for 26.39% of the samples. Conversely, the new algorithm does not activate any false positives, resulting in nearly perfect precision in detecting landings.

### 4.3. Analysis of the Flight Time

**Figure 9:**
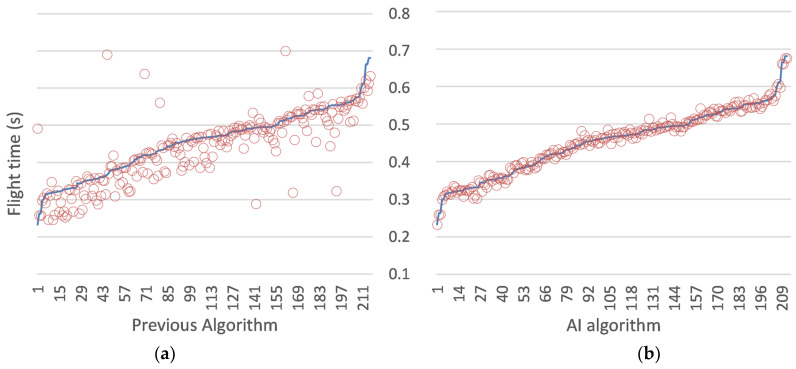
Comparison between the predicted flight times by algorithms and ground truth. Blue line: ground truth values; red circles: values predicted by the algorithm: (**a**) previous algorithm; (**b**) AI algorithm.

When comparing the values obtained by both algorithms in the detection and calculation of flight time study with the data provided by the same samples measured in a commercial jump mat [17] (ground truth), a significant improvement between the two algorithms is observed, as shown in Figure 9. In the previous algorithm without the use of AI, an average error of 31.6 ms with a standard deviation of 53.3 ms is obtained, whereas in the AI-based algorithm, an average error of 7 ms with a standard deviation of 9 ms is obtained. The temporal error introduced by our algorithm results in a negligible error in the measurement of the jump height. For instance, in a typical 30 cm jump, the athlete’s flight time is 495 ms, and adding an increment of 7 ms introduces a change of 8.5 mm. This error is negligible and falls below the minimum expected change that a coach would look for to conclude that there was a significant change in the jump height. The proposed system outperforms the typical error of existing methods in the literature. Different instruments based on the timekeeping of take-off and landing events show a variety of errors, which are greater than the proposed system. For instance, jump mats typically exhibit around 1.2 cm error [9], whereas photocells produce a 1.3 cm error [10] and smartphone apps introduce a 1.1 cm error [12], considering that the latter needs human observation to retrieve the outcome. These errors are substantially higher than the error introduced by our AI-based algorithm, which is negligible at around 8.5 mm for a typical 30 cm jump. The proposed system’s superior accuracy can be attributed to its utilization of AI-based learning for jump landing recognition, which makes it more robust to noise compared to the traditional methods that solely rely on signal energy analysis. As we can see, the system is much more robust to noise as it utilizes AI-based learning for jump landing recognition, whereas the previous algorithm solely relied on studying the signal’s energy, leading to inaccuracies and false positives when noise was added to the samples.

### 4.4. Analysis of Synthetically Created Jumps

Building upon the insights gleaned from previous studies, which underscored the system’s effectiveness, we strove to deepen our understanding through further exploration. To this end, we conceived a novel approach: the generation of synthetic jumps derived from existing data. By combining both take-off and landing events and introducing arbitrary time intervals, we aimed to expand the scope of our investigation.

**Figure 10:**
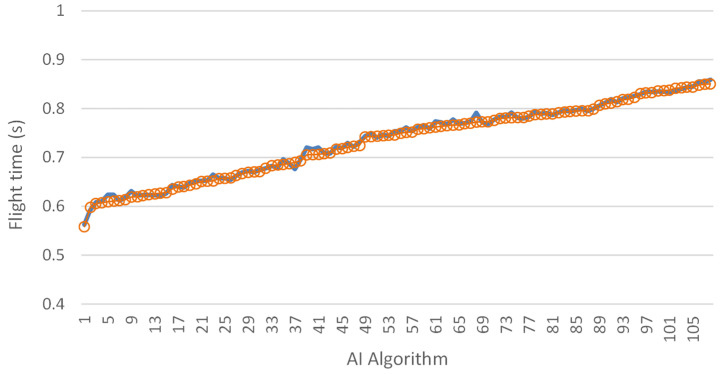
Comparison of flight times between the created samples and AI algorithm predictions. Blue line: ground truth values; red circles: values predicted by the algorithm.

Building on our previous work, we created synthetic jumps by precisely combining take-off and landing segments from existing audio recordings. These synthetic jumps were crafted with the flexibility to introduce arbitrary time intervals between the take-off and landing events. Such a methodology not only allows for the exploration of diverse scenarios but also provides a means to test the algorithm’s robustness across various temporal configurations. This process ensures that each synthetic jump is crafted to mirror real-world dynamics, thereby enriching our dataset and enhancing the comprehensiveness of our analysis. Subsequently, these synthetic jumps undergo the same rigorous evaluation process as their real-world counterparts, facilitating a comprehensive comparison of algorithmic performance across synthetic and real-world scenarios. The results of this analysis can be found in Figure 10. Through this innovative approach, we aimed to glean deeper insights into the system’s capabilities and limitations, thereby paving the way for further refinement and optimization. By meticulously examining the performance of our algorithm in synthetic scenarios, we endeavored to fortify its efficacy and reliability across diverse real-world applications.

As a future work, there is potential in integrating this algorithm into mobile applications tailored for devices such as smartphones and tablets. Such an endeavor would enable users to engage in the real-time tracking of jump events during athletic endeavors through their mobile devices. Efforts can be directed towards optimizing the algorithm’s performance on devices with constrained computational resources, facilitating its deployment across a broad spectrum of mobile platforms.

### 4.5. Practical Applications

The proposed audio-based system for measuring jump height offers significant practical implications for sports science professionals and athletes alike. The robustness and accuracy of the AI-based algorithm provide a reliable and portable tool for monitoring and assessing athletic performance in various environments. For sports science professionals, this system presents a valuable opportunity to conduct comprehensive evaluations of an athlete’s lower-body power and neuromuscular coordination. By accurately measuring jump height, coaches and trainers can gain insights into an athlete’s functional capabilities, enabling them to tailor training programs and track progress over time. The system’s ability to operate effectively in challenging acoustic environments, such as crowded gyms or outdoor settings, further enhances its practical utility. Athletes can benefit from the system’s portability and ease of use. Integrated into a mobile application, this technology empowers athletes to self-monitor their jump performance, fostering self-awareness and enabling them to identify areas for improvement. Additionally, the system’s non-intrusive nature eliminates the need for specialized equipment, allowing athletes to conduct jump assessments in their preferred training environments without disrupting their routines. Furthermore, the system’s applicability extends beyond individual athlete assessments. Sports science professionals can use this technology to conduct large-scale screening and talent identification programs, evaluating numerous athletes efficiently and accurately. This capability can streamline the scouting process and aid in the early identification of promising athletes, facilitating targeted development and resource allocation.

The proposed system demonstrates significant scalability and potential for commercialization or widespread adoption in sports training and performance monitoring. Its ability to function effectively on mobile devices, combined with its robustness and ease of use, makes it an attractive solution for various stakeholders in the sports industry. From amateur athletes and local sports clubs to professional teams and organizations, this system can be readily integrated into existing training and monitoring protocols, providing a cost-effective and accessible tool for performance evaluation.

Overall, the proposed audio-based system for measuring jump height represents a significant advancement in sports science technology, offering practical benefits to both professionals and athletes. By providing a reliable, portable, and accessible tool for performance evaluation, this system empowers stakeholders to make informed decisions, optimize training regimens, and ultimately enhance athletic development and achievement.

## 5. Conclusions

In this study, we have developed an innovative approach for measuring flight time in jump events in noisy acoustic environments. Our approach relies on the implementation of a convolutional neural network (CNN) for audio event detection in the jumps, both landings and take-offs. This novel approach addresses the limitations of previous algorithms, which were prone to failure when confronted with audio samples containing high levels of background noise and impulsive interference.

Furthermore, the selection of robust features was crucial in enabling the comprehensive analysis of jump events amidst noisy environments. These features have provided insights into both the spectral components of the events and the temporal variations in predominantly impulsive noises, thereby enhancing our ability to discern relevant event signatures.

We demonstrated the efficacy of convolutional neural networks (CNNs) as efficient deep neural networks (DNNs) for audio event detection, particularly in identifying impulsive events like jump take-offs and landings. Leveraging their machine learning capabilities and inherent robustness to noise, CNNs exhibit remarkable proficiency in distinguishing between background noise and target events of interest, thus offering valuable insights into the temporal dynamics and spectral characteristics of these occurrences.

The algorithm’s efficacy is validated through rigorous experimentation, employing robust methodologies and thorough validation procedures. By introducing noise and synthesizing audio samples, the algorithm demonstrates resilience and adaptability across diverse environments. A comparative analysis with previous algorithms revealed significant enhancements in precision and robustness, notably reducing both calculation errors for flight time estimation and false positive rates compared to previous approaches. These findings underscore the algorithm’s effectiveness in analyzing jump events amidst noisy acoustic environments and highlight its potential for practical deployment in various settings, such as sports events, biomechanics research, and athlete training programs. Ultimately, the proposed algorithm represents a significant leap forward in the accurate measurement of jump flight times, offering a reliable and robust solution that overcomes the limitations of previous approaches, particularly in challenging acoustic environments.

With its exceptional performance in mitigating the challenges posed by noisy environments and impulsive interference, this innovative algorithm facilitates the way for more precise and reliable assessment of athlete performance, enabling coaches, researchers, and athletes to unlock new insights and optimize training strategies.

## Figures and Tables

**Table 1:** Results of the landing evaluation.

K-Fold	Precision (%)	Recall (%)	F1-Score (%)
1	0.925	0.974	0.949
2	0.951	0.975	0.963
3	0.999	0.891	0.943
4	1.000	1.000	1.000
5	0.974	0.974	0.974
Mean	0.970	0.963	0.966

**Table 2:** Results of the take-off evaluation.

K-Fold	Precision (%)	Recall (%)	F1-Score (%)
1	0.974	0.974	0.974
2	0.951	0.951	0.951
3	0.960	0.904	0.931
4	0.965	0.980	0.971
5	0.904	0.999	0.949
Mean	0.951	0.962	0.956

## Data Availability

The data presented in this study are available on reasonable request from the corresponding author.
